# Supplementary material for: Molecular and Serological Detection of Leishmania spp. in Mediterranean Wild Carnivores and Feral Cats: Implications for Wildlife Health and One Health Surveillance
Source: Animals (Basel). 2025 Sep 20;15(18):2751. doi: 10.3390/ani15182751 (PMC12466643; doi:10.3390/ani15182751)
Supplement: Supplementary file 1 [file animals-15-02751-s001.zip › animals-3796602-supplementary.pdf]

Specie	Reference	Estimated Date of Death	Age	Sex	Origin	Latitude	Longitude	ELISA tested	Result
Eurasian badger	Mm20001	2020	Nd	Nd	Nd	39,55984372	-1,152086033	yes	-
Eurasian badger	Mm21001	28/1/21	Nd	Nd	Nd	39,77549	-1,0456	-	-
Eurasian badger	Mm21002	4/2/21	juvenile	male	Cullera	39,18328	-0,26711	-	-
Eurasian badger	Mm21003	4/2/21	adult	female	Otos	38,87097	-0,4482	-	-
Eurasian badger	Mm21004	4/3/21	adult	male	Tuejar	39,77549	-1,0456	-	-
Eurasian badger	Mm21005	4/3/21	adult	male	Villar del Arzobispo	39,70083	-0,78917	yes	positive (qPCR)
Eurasian badger	Mm21006	30/4/21	adult	female	Andilla	39,83447	-0,81489	yes	-
Eurasian badger	Mm21009	10/9/21	adult	female	Las Cuevas (Utiel)	39,61807	-1,2544	yes	-
Eurasian badger	Mm21010	22/11/21	adult	female	Chelva, A3	39,47775	-0,72397	yes	-
Eurasian badger	Mm22001	19/1/22	adult	female	Casinos	39,695	-0,72111	yes	-
Eurasian badger	Mm22002	28/1/22	adult	male	Utiel	39,61807	-1,2544	-	-
Eurasian badger	Mm22003a	28/1/22	adult	male	Requena	39,48435	-1,06423	yes	-
Eurasian badger	Mm22004	2/2/22	adult	male	Chelva	39,74738	-0,98952	yes	-
Eurasian badger	Mm22005	4/5/22	adult	male	Chulilla	39,67894	-0,88024	yes	-
Eurasian badger	Mm23001	27/4/23	adult	male	Villanueva de Alcolea	40,26806	-0,08306	yes	-
Eurasian badger	Mm23002	27/4/23	adult	female	Chulilla	39,64141	-0,88354	yes	-
Eurasian badger	Mm23003	27/4/23	adult	female	Ares del Maestrat	40,42568	-0,13858	yes	-
Eurasian badger	Mm24001	15/2/24	Nd	female	Canet lo Roig	40,54884	0,24423	yes	-
Eurasian badger	Mm24002	1/3/24	adult	female	Lliria	39,64111	-0,59694	yes	-
Eurasian badger	Mm24003	1/3/24	adult	male	Requena	39,50513	-1,10601	yes	-
Eurasian badger	Mm24004	30/7/24	adult	male	Canet lo Roig	40,54884	0,24423	yes	-
Eurasian badger	Mm24005	31/7/24	adult	male	Vilanueva de Alcolea	40,22932	0,08094	yes	-

Specie	Reference	Estimated Date of Death	Age	Sex	Origin	Latitude	Longitude	ELISA tested	Result
Eurasian badger	Mm24007	1/10/24	adult	female	Villar del Arzobispo	39,70056	-0,82389	yes	-
Eurasian badger	Mm25001	14/2/25	adult	male	Belgida	38,86057	-0,47835	yes	-
Eurasian badger	Mm25002	14/2/25	adult	male	Ribarroja Turia	39,53871	-0,57321	yes	-
Eurasian badger	Mm25003	14/2/25	adult	female	Requena	39,48435	-1,06423	yes	-
Eurasian badger	Mm25004	14/2/25	adult	female	Albocasser	40,37448	-0,05684	yes	-
Eurasian badger	Mm25005	8/5/25	adult	Nd	Pau (Jávea)	38,77519	0,13462	yes	-
Eurasian badger	Mm25006	8/5/25	adult	male	Villar del Arzobispo	39,70222	-0,76583	-	-
European wildcat	Fs21001	14/12/20	Nd	Nd	Venta del Moro	39,50609	-1,3491	yes	-
European wildcat	Fs21002	21/4/21	adult	female	Venta del Moro	39,50609	-1,3491	-	-
European wildcat	Fs21003	23/10/22	adult	female	Alpera (Bonete)	38,96392	-1,24104	-	-
Eurasian otter	Ll21001	25/8/21	adult	male	Beniganim	38,93889	-0,45683	yes	-
Eurasian otter	Ll21003	6/8/21	adult	female	Almassora	39,92806	-0,02944	yes	-
American mink	NV-2101	1/2/21	Nd	male	Rio Palancia (Segorbe)	39,84334	-0,46849	-	-
American mink	NV-2102	21/1/21	Nd	female	Rio Mijares (Fanzara)	40,01746	-0,30863	-	-
American mink	NV-2103	20/11/20	juvenile	female	Rio Mijares (Arañuel)	40,07208	-0,47749	-	-
American mink	NV-2104	28/1/21	adult	male	Rio Palancia (Soneja)	39,82117	-0,42562	-	-
American mink	NV-2105	28/1/21	adult	male	Rio Palancia (Soneja)	39,82277	-0,43918	-	-
American mink	NV-2106	29/1/21	adult	male	Rio Palancia (Segorbe)	39,84334	-0,46849	-	-
American mink	NV-2107	23/11/20	juvenile	female	Rio Mijares (Onda)	39,98541	-0,22282	-	positive (qPCR)

Specie	Reference	Estimated Date of Death	Age	Sex	Origin	Latitude	Longitude	ELISA tested	Result
American mink	NV-2108	20/11/20	adult	female	Rio Mijares (Fanzara)	40,01746	-0,30863	-	-
American mink	NV-2109	19/1/21	adult	male	Rio Mijares (Onda)	39,98088	-0,16597	-	-
American mink	NV-2110	18/11/20	Nd	male	Rio Palancia (Jerica)	39,91636	-0,57852	-	-
American mink	NV-2111	14/1/21	Nd	male	Rio Mijares (Toga)	40,04283	-0,39195	-	-
American mink	NV-2112	14/12/20	Nd	female	Rio Palancia (Navajas)	39,86792	-0,48997	-	-
American mink	NV-2113	2/2/21	Nd	male	Rio Palancia (Soneja)	39,81417	-0,41519	-	-
American mink	Nv24001	21/2/22	adult	male	Rio Palancia (Jerica)	39,88758	-0,5362	-	-
American mink	Nv24002	17/3/22	adult	male	Rio Palancia (Navajas)	39,87938	-0,50549	yes	-
American mink	Nv24003	4/3/22	adult	male	Rio Mijares (Cirat)	40,05758	-0,47177	yes	-
American mink	Nv24004	18/2/22	adult	male	Rio Palancia (Soneja)	39,82117	-0,42562	yes	-
American mink	Nv24005	13/4/22	adult	female	Rio Mijares (Cirat)	40,05821	-0,43088	yes	-
American mink	Nv24006	10/3/22	adult	male	Rio Mijares (Onda)	39,98088	-0,16597	yes	-
American mink	Nv24007	25/2/22	adult	male	Rio Palancia (Soneja)	39,81417	-0,41519	yes	-
American mink	Nv24008	22/2/22	adult	female	Rio Palancia (Jerica)	39,89542	-0,54961	yes	-
American mink	Nv24009	22/2/22	adult	female	Rio Palancia (Jerica)	39,91636	-0,57852	yes	-
American mink	Nv24010	3/3/22	adult	female	Rio Mijares (Fanzara)	40,00716	-0,30682	yes	-

Specie	Reference	Estimated Date of Death	Age	Sex	Origin	Latitude	Longitude	ELISA tested	Result
American mink	Nv24011	18/3/22	adult	male	Rio Palancia (Jerica)	39,89542	-0,54961	-	-
American mink	Nv24012	17/3/22	adult	female	Rio Palancia (Segorbe)	39,85324	-0,47486	-	-
American mink	Nv24013	23/2/22	adult	male	Rio Palancia (Navajas)	39,87938	-0,50549	yes	-
Cat	Fc23037	2023	adult	male	Cofrentes	39,21552085	-1,060190614	-	-
Cat	Fc23038	2023	adult	female	Cofrentes	39,22105957	-1,056160043	-	-
Cat	Fc23039	2023	adult	female	Cofrentes	39,2174511	-1,063741582	yes	-
Cat	Fc23053	2024	adult	male	Venta del Moro	39,52523823	-1,40348715	-	-
Cat	Fc23055	2024	adult	female	Cofrentes	39,20702071	-1,057226825	-	-
Cat	Fc23060	2024	adult	female	Sinarcas	39,65922273	-1,150491496	-	-
Cat	Fc23068	2024	adult	male	Venta del Moro	39,51552739	-1,407171215	-	-
Cat	Fc23077	2024	adult	male	Venta del Moro	39,51957036	-1,404313111	-	-
Cat	Fc23081	2024	adult	male	Venta del Moro	39,51595106	-1,404826108	yes	-
Cat	Fc23085	2024	adult	male	Venta del Moro	39,51800238	-1,402909252	-	-
Cat	Fc23092	2024	adult	male	Venta del Moro	39,51549984	-1,409439267	yes	-
Cat	Fc23095	2024	adult	male	Venta del Moro	39,51001405	-1,403435438	-	-
Cat	Fc23097	2024	adult	female	Venta del Moro	39,51933757	-1,396610625	-	-

Specie	Reference	Estimated Date of Death	Age	Sex	Origin	Latitude	Longitude	ELISA tested	Result
Cat	Fc23098	2024	adult	male	Venta del Moro	39,50614441	-1,403942762	-	-
Cat	Fc23100	2025	adult	male	Venta del Moro	39,51940673	-1,405873203	-	-
Cat	Fc23101	2025	adult	male	Venta del Moro	39,52531204	-1,393830785	-	-
Cat	Fc23102	2025	adult	female	Venta del Moro	39,52268953	-1,392074355	-	-
Cat	Fc23103	2025	adult	female	Venta del Moro	39,52449524	-1,392743088	yes	-
Cat	Fc23104	2025	adult	male	Venta del Moro	39,51778494	-1,390260304	-	-
Cat	Fc23105	2025	adult	female	Venta del Moro	39,52283886	-1,399852836	yes	-
Cat	Fc23106	2025	adult	male	Venta del Moro	39,50659852	-1,391836308	yes	-
Cat	Fc23108	2025	adult	male	Venta del Moro	39,5233409	-1,394751145	yes	-
Cat	Fc23109	2025	adult	female	Venta del Moro	39,52555245	-1,399272322	yes	-
Cat	Fc23110	2025	adult	female	Venta del Moro	39,52250737	-1,393355436	-	-
Cat	Fc23111	2025	adult	female	Venta del Moro	39,51392129	-1,397819282	yes	-
Cat	Fc23113	2025	adult	female	Venta del Moro	39,51779107	-1,400598699	-	-
Cat	Fc23116	2025	adult	female	Camporrobles	39,64183544	-1,291968704	yes	-
Cat	Fc23117	2025	adult	male	Camporrobles	39,6541068	-1,308556214	-	-
Cat	Fc23119	2025	adult	male	Camporrobles	39,65272421	-1,297370137	-	-

Specie	Reference	Estimated Date of Death	Age	Sex	Origin	Latitude	Longitude	ELISA tested	Result
Cat	Fc23120	2025	adult	female	Camporrobles	39,64037784	-1,305923076	yes	-
Cat	Fc23121	2025	adult	male	Camporrobles	39,65388644	-1,298086906	yes	-
Cat	Fc23122	2025	adult	female	Venta del Moro	39,52208328	-1,400054262	-	-
Cat	Fc23123	2025	adult	male	Venta del Moro	39,5221769	-1,398434182	-	-
Cat	Fc23124	2025	adult	female	Venta del Moro	39,52185537	-1,395900491	yes	-
Common genet	Gg21002	20/2/21	juvenile	female	Nd	39,53352646	-1,228171785	-	-
Common genet	Gg21003	20/2/21	adult	male	La Vall D'Uxó	39,83065	-0,20565	yes	-
Common genet	Gg21006	7/1/21	adult	female	Cabanes	40,15366	0,05034	yes	-
Common genet	Gg21007	2021	adult	male	Nd	39,5068776	-0,970509123	yes	-
Common genet	Gg21008	30/9/21	adult	female	Nd	39,70605756	-0,996239242	-	-
Common genet	Gg21009	21/7/21	adult	male	Domeño	39,70389	-0,92944	-	-
Common genet	Gg21010	2021	adult	female	Nd	39,79656645	-1,229105983	yes	-
Common genet	Gg21011	13/3/21	adult	male	Alborache	39,39477	-0,76781	yes	-
Common genet	Gg21012	1/12/21	adult	female	El Saler	39,67107148	-1,259637757	yes	-
Common genet	Gg22001a	2022	adult	male	Nd	39,60346573	-0,995459352	yes	-
Common genet	Gg22002	13/2/22	adult	male	Vallada	38,90737	-0,68737	-	-
Common genet	Gg22003	3/3/22	adult	male	Gátova	39,7728	-0,52647	-	-
Common genet	Gg22004	21/4/22	adult	male	Bicorp	39,12606	-0,78675	yes	-

Specie	Reference	Estimated Date of Death	Age	Sex	Origin	Latitude	Longitude	ELISA tested	Result
Common genet	Gg22005	5/6/22	adult	male	Caudete de las Fuentes	39,54293	-1,28946	-	-
Common genet	gg23001	8/3/23	adult	female	Jávea	38,77519	0,13462	yes	-
Common genet	Gg23003	2023	adult	Nd	Nd	39,66694457	-1,050599826	yes	-
Common genet	Gg24001	4/1/24	adult	male	Benicull de Xuquer	39,18867	-0,3836	-	-
Common genet	Gg24002	19/5/23	adult	male	Valencia	39,54528	-0,46222	-	-
Common genet	Gg24003	4/5/24	adult	male	Cabanes	40,15366	0,05034	-	-
Common genet	Gg25001	7/1/25	adult	male	Villar del Arzobispo	39,70138	-0,77762	yes	-
Common genet	Gg25002	3/2/25	adult	female	Moncada	39,56716	-0,41281	yes	-
Common genet	Gg25003	2025	adult	male	Llíria	39,63833	-0,61722	yes	-
Common genet	Gg25004	1/3/25	adult	male	Requena	39,48435	-1,06423	yes	-
Stone marten	Mf21001	6/2/21	adult	male	Vilanova de Alcolea	40,22932	0,08094	yes	-
Stone marten	Mf21005	11/1/21	adult	male	Utiel	39,61807	-1,2544	yes	-
Stone marten	Mf21006	7/3/21	adult	female	Dos Aguas	39,29107	-0,8019	yes	-
Stone marten	Mf21007	23/4/21	adult	male	Sinarcas	39,71975	-1,23702	-	-
Stone marten	Mf21008	27/5/21	adult	female	Jarafuel	39,12686	-1,06978	-	-
Stone marten	Mf21009	25/8/21	adult	male	Utiel	39,61807	-1,2544	yes	-
Stone marten	Mf21010	2021	adult	female	Nd	39,76964995	-1,182494356	yes	-
Stone marten	Mf21012	13/9/21	adult	male	Vallat (CV20)	40,02098	-0,3283	yes	-
Stone marten	Mf21013	2021	adult	female	Puebla del Duc cv60	38,88782	-0,4399	yes	-
Stone marten	Mf21014	8/9/21	adult	male	La Yesa	39,85598	-0,91735	yes	-
Stone marten	Mf21015	23/12/21	adult juvenile	male	Ayora	39,07434	-1,05427	yes	-

Specie	Reference	Estimated Date of Death	Age	Sex	Origin	Latitude	Longitude	ELISA tested	Result
Stone marten	Mf21016	23/12/21	juvenile	female	Losa del Obispo	39,69778	-0,86861	yes	-
Stone marten	Mf21017	23/12/21	Nd	male	Nd	39,76607013	-1,134006911	yes	-
Stone marten	Mf21018	3/12/21	adult	female	Jarafuel	39,13333	-1,075	yes	-
Stone marten	MF22001a	9/12/21	adult	male	Venta del Moro	39,48692	-1,38055	yes	-
Stone marten	MF22005	26/4/22	adult	male	Corbera	39,16473	-0,3594	yes	-
Stone marten	Mf23001	10/2/23	adult	male	CV35	39,65415904	-1,082328049	yes	-
Stone marten	Mf23002	17/3/23	adult	male	Morella	40,62378	-0,094	yes	-
Stone marten	Mf23004	18/11/23	adult	male	Villar del Arzobispo	39,70083	-0,78917	yes	-
Stone marten	Mf24001	27/12/22	adult	female	Vall d'Alba	40,16634	-0,0055	-	-
Stone marten	Mf24002	12/5/23	adult	female	Simat de la Valldigna	39,03666	-0,30454	-	-
Stone marten	Mf24003	28/4/24	adult	female	Vallanca	40,06065	-1,32666	-	-
Stone marten	Mf24004	2024	adult	male	Chera	39,5989	-0,96262	-	-
Stone marten	Mf24005	3/4/24	adult	male	Els Ports (Morella)	40,62378	-0,094	-	-
Stone marten	Mf24006	2024	adult	male	Vilanova D'Alcolea	40,22361	0,05	yes	-
Stone marten	Mf24007	22/11/24	adult	male	Alzira	39,16291	-0,45629	yes	-
Stone marten	Mf25001	24/2/25	adult	male	Casinos	39,70222	-0,76583	yes	-
Stone marten	Mf25002	5/12/24	adult	male	CV35	39,55984372	-1,152086033	-	-
Stone marten	Mf25003	30/12/24	adult	female	Sinarcas	39,73855	-1,22913	yes	-
Stone marten	Mf25004	1/8/24	adult	female	Tirig	40,43079	0,08173	yes	-
Stone marten	Mf25005	26/5/25	adult	male	Paterna	39,54528	-0,46222	yes	positive (qPCR)
Red fox	Vv19002	2019	adult	male	Vilafranca del Cid	40,43339	-0,2498	yes	-

Specie	Reference	Estimated Date of Death	Age	Sex	Origin	Latitude	Longitude	ELISA tested	Result
Red fox	Vv19003	2019	juvenile	male	Nd	39,77802224	-1,15546898	yes	-
Red fox	Vv19004	2019	adult	female	Casinos	39,66972	-0,67389	yes	positive (qPCR)
Red fox	Vv19005	2019	adult	male	RVCMC	39,17863	-0,93606	yes	-
Red fox	Vv19006	2019	adult	female	Nd	39,75338013	-1,010721192	yes	-
Red fox	Vv19007	2019	adult	male	Nd	39,57887236	-1,10587924	yes	-
Red fox	Vv19008	2019	juvenile	female	Casinos	39,695	-0,72111	yes	-
Red fox	Vv19009	2019	adult	male	Lìria	39,63222	-0,58028	yes	-
Red fox	Vv19010	2019	adult	female	Villar del Arzobispo	13,8	40,5	yes	positive (ELISA)
Red fox	Vv19011	2019	adult	female	LÌria	39,64861	-0,63611	yes	-
Red fox	Vv19012	2019	Nd	male	Villar del Arzobispo	39,70083	-0,78917	yes	-
Red fox	Vv19013	2019	Nd	male	Casinos	39,695	-0,72111	yes	-
Red fox	Vv20001	2020	adult	male	Villar del Arzobispo	39,70138	-0,77762	yes	-
Red fox	Vv20002	2020	adult	male	Meliana	39,52291	-0,35706	yes	-
Red fox	Vv20003	6/3/20	Nd	male	Nd	39,79957847	-1,038650368	yes	-
Red fox	Vv20004	2020	Nd	male	Mas Camarena	39,53278	-0,44639	yes	-
Red fox	Vv20005	2020	juvenile	female	Villar del Arzobispo	39,70056	-0,81222	yes	-
Red fox	Vv20008	11/11/20	Nd	female	CV35	39,56462645	-1,207226695	yes	Positive (ELISA)
Red fox	Vv21001	24/1/21	adult	male	Manises	39,68132	-0,54367	yes	Positive (qPCR + ELISA)
Red fox	Vv21002	17/12/20	adult	female	Paterna	39,54528	-0,46222	-	-
Red fox	Vv21005	2021	juvenile	male	Nd	39,78414138	-1,208789859	yes	-
Red fox	Vv21007	2021	juvenile	female	AlgemesÌ	39,20428	-0,42834	-	-
Red fox	Vv21010	6/5/21	adult	male	Jávea	38,77519	0,13462	yes	-
Red fox	Vv21014	6/7/21	adult	female	Calpe	38,65696	0,03685	-	-
Red fox	Vv21018	29/7/21	adult	male	Cabanes	40,15366	0,05034	-	-

Specie	Reference	Estimated Date of Death	Age	Sex	Origin	Latitude	Longitude	ELISA tested	Result
Red fox	Vv21020	13/8/21	adult	female	Benisanó	39,60934	-0,56517	yes	-
Red fox	Vv21021	15/8/21	adult	female	CV35	39,76395568	-1,002593074	yes	-
Red fox	Vv21022	8/3/21	adult	male	Casinos	39,69111	-0,71056	yes	-
Red fox	Vv21023	4/9/21	adult	male	Paterna	39,54972	-0,47222	yes	positive (qPCR)
Red fox	Vv21024	23/9/21	adult	male	Soneja	39,81908	-0,41101	yes	-
Red fox	Vv21025	9/10/21	adult	female	Casinos	39,695	-0,72111	yes	-
Red fox	Vv21026	13/10/21	adult	male	Utiel	39,56363	-1,20647	yes	-
Red fox	Vv21030	28/11/21	adult	female	Benaguacil	39,60346	-0,58698	yes	-
Red fox	Vv21031	2021	Nd	Nd	CV35	39,67775881	-1,222473368	yes	-
Red fox	Vv22002	17/1/22	adult	female	Cabanes	40,15366	0,05034	yes	positive (qPCR)
Red fox	Vv22003	17/1/22	adult	male	Pobla de Vallbona	39,57528	-0,51944	yes	-
Red fox	Vv22004	17/1/22	adult	male	Villar del Arzobispo	39,70056	-0,82389	yes	-
Red fox	Vv22008	30/1/22	adult	female	Alpera	38,96392	-1,24104	yes	-
Red fox	Vv22010	3/2/22	juvenile	male	Villar del Arzobispo	39,73484	-0,8301	yes	-
Red fox	VV22027	7/10/22	juvenile	female	Villar del Arzobispo	39,70138	-0,77762	-	-
Red fox	VV22028	21/11/22	adult	male	Chiva	39,47775	-0,72397	-	-
Red fox	VV22029	23/10/22	adult	Nd	Villar del Arzobispo	39,70138	-0,77762	-	-
Red fox	VV22030	9/5/22	juvenile	female	Villar del Arzobispo	39,69833	-0,84694	-	-
Red fox	VV22031	25/11/22	juvenile	male	Utiel	39,61807	-1,2544	-	-
Red fox	Vv22032	27/11/22	juvenile	female	Casinos	39,69111	-0,71056	-	-
Red fox	Vv22033	1/11/22	adult	Nd	Godelleta	39,42209	-0,68142	-	-
Red fox	Vv23001	2023	adult	male	CV35	39,56874694	-1,003871612	yes	-
Red fox	Vv23003	13/1/23	adult	male	Requena	39,48111	-1,14944	yes	-
Red fox	Vv23004	2023	adult	male	Canals	38,9587	-0,56816	yes	-
Red fox	Vv23005	2023	adult	male	Villar del Arzobispo	39,73484	-0,8301	yes	-

Specie	Reference	Estimated Date of Death	Age	Sex	Origin	Latitude	Longitude	ELISA tested	Result
Red fox	Vv23006	2023	adult	male	Villar del Arzobispo	39,69833	-0,84694	yes	-
Red fox	Vv23008	25/1/23	adult	female	Villar del Arzobispo	39,69833	-0,84694	yes	-
Red fox	Vv23009	20/3/23	adult	female	Ara de los Olmos	39,60889	-0,56583	yes	-
Red fox	Vv23010	26/1/23	adult	female	San Antonio de Benageber	39,55806	-0,49278	yes	-
Red fox	Vv23011	25/2/23	adult	male	Pobla de Vallbona	39,60132	-0,54448	yes	-
Red fox	Vv23013	21/4/23	adult	female	Jávea	38,77519	0,13462	yes	-
Red fox	Vv23014	4/2/23	adult	female	Jávea	38,77519	0,13462	yes	-
Red fox	Vv23015	28/3/23	Nd	Nd	Jávea	38,77519	0,13462	yes	-
Red fox	Vv23016	20/4/23	Nd	male	Jávea	38,77519	0,13462	yes	-
Red fox	Vv23017	19/4/23	adult	male	Jávea	38,77519	0,13462	yes	-
Red fox	Vv23018	19/5/23	juvenile	male	Villar del Arzobispo	39,69833	-0,84694	yes	-
Red fox	Vv23019	2023	adult	male	CV35	39,58184381	-1,270527334	yes	-
Red fox	Vv24001	30/12/22	juvenile	female	Bétera	39,59998	-0,45652	yes	-
Red fox	Vv24002	17/2/24	adult	male	Vergel	38,86642	-0,02445	yes	positive (qPCR)
Red fox	Vv24003	20/12/23	adult	male	Mas Camarena	39,54528	-0,46222	yes	-
Red fox	Vv24004	28/12/23	adult	female	Casinos	39,69111	-0,71056	yes	-
Red fox	Vv24005	27/12/23	adult	male	Muro de Alcoy	38,78185	-0,42754	yes	Positive (ELISA)
Red fox	VV24008	7/7/24	adult	female	Villar del Arzobispo	39,73484	-0,8301	yes	-
Red fox	Vv24009	26/6/24	juvenile	male	Godelleta	39,42209	-0,68142	yes	-
Red fox	VV24010	27/5/24	juvenile	male	La Salzadella	40,51036	0,19464	yes	-
Red fox	Vv24012	25/7/24	adult	female	Pobla de Vallbona	39,58056	-0,52917	yes	positive (qPCR)
Red fox	Vv24013	7/7/24	adult	male	Cabanes	40,15366	0,05034	yes	-
Red fox	Vv24014	28/5/24	juvenile	female	Jávea	38,77519	0,13462	yes	-
Red fox	Vv24015	10/7/24	juvenile	female	Sagunto	39,72545	-0,21718	yes	-

Specie	Reference	Estimated Date of Death	Age	Sex	Origin	Latitude	Longitude	ELISA tested	Result
Red fox	VV24016	1/8/24	adult	male	Godelleta	39,42209	-0,68142	yes	-
Red fox	Vv24017	9/8/24	juvenile	female	Godelleta	39,42209	-0,68142	yes	positive (qPCR)
Red fox	Vv24019	30/8/24	adult juvenile	male	Ara de los Olmos	39,60889	-0,56583	yes	-
Red fox	Vv24021	2/8/24	juvenile	female	Villar del Arzobispo	39,69833	-0,84694	yes	-
Red fox	Vv24022	2024	adult	male	CV35	39,73220855	-1,029668356	yes	-
Red fox	Vv24023	1/10/24	adult	female	San Antonio de Benageber	39,55278	-0,48306	yes	-
Red fox	Vv24024	1/3/24	adult	female	CV35	39,75423595	-0,943159577	yes	-
Red fox	Vv24025	2024	adult	female	Denia	38,83859	0,09273	yes	-
Red fox	Vv24026	12/10/24	adult	female	L'Alcora	40,07061	-0,20329	yes	-
Red fox	Vv24027	2024	adult	male	Nd	39,75834697	-1,097914727	yes	-
Red fox	Vv25001	24/12/24	adult	male	Villar del Arzobispo	39,70056	-0,80083	yes	-
Red fox	Vv25003	22/1/25	adult	female	Jávea	38,76955	0,16191	yes	-
Red fox	Vv25004	22/1/25	adult	male	Jávea	38,76955	0,16191	yes	positive (qPCR)
Red fox	Vv25005	2025	adult	male	Bélgida	38,86057	-0,47835	yes	-
Red fox	Vv25006	8/2/25	adult	female	Bufalí	38,81877	-0,48358	yes	-
Red fox	Vv25007	8/2/25	adult	female	Bufalí	38,81877	-0,48358	yes	-
Red fox	Vv25008	8/2/25	adult	female	Bufalí	38,81877	-0,48358	yes	positive (ELISA)
Red fox	Vv25009	8/2/25	adult	male	Bufalí	38,81877	-0,48358	yes	-
Red fox	Vv25010	8/2/25	adult	female	Bufalí	38,81877	-0,48358	yes	-
Red fox	Vv25011	8/2/25	adult	female	Bufalí	38,81877	-0,48358	yes	-
Red fox	Vv25012	8/2/25	adult	male	Bufalí	38,81877	-0,48358	yes	-
Red fox	Vv25013	8/2/25	adult	female	Bufalí	38,81877	-0,48358	yes	-
Red fox	Vv25014	10/4/25	adult	female	Jávea	38,77519	0,13462	-	-
Red fox	Vv25015	8/2/25	adult	female	Bufalí	38,81877	-0,48358	-	-
Red fox	Vv25016	14/4/25	adult	male	Godelleta	39,42209	-0,68142	yes	positive (qPCR)
Red fox	Vv25017	2025	adult	female	CV35	39,5459954	-1,24098012	yes	-

Specie	Reference	Estimated Date of Death	Age	Sex	Origin	Latitude	Longitude	ELISA tested	Result
Red fox	Vv25018	1/3/25	adult	female	Jávea	38,77519	0,13462	yes	-
Red fox	Vv25019	28/2/25	adult	male	Requena	39,48435	-1,06423	-	positive (qPCR)
